# Scaling COVID-19 rates with population size in the United States

**DOI:** 10.1098/rsif.2024.0839

**Published:** 2025-03-26

**Authors:** Austin R. Cruz, Brian J. Enquist, Joseph R. Burger

**Affiliations:** ^1^ Department of Ecology & Evolutionary Biology, The University of Arizona, Tucson, AZ, USA; ^2^ Santa Fe Institute, Santa Fe, NM, USA; ^3^ University of Kentucky, Lexington, KY, USA

**Keywords:** scaling, COVID-19, epidemiology, scaling laws, allometry

## Abstract

Using county-level data from the United States, we assessed allometric scaling relationships of coronavirus disease (COVID-19) cases, deaths and age structure within and across the first four major waves of the pandemic (wild-type, alpha, delta, omicron). Results generally indicate that the burden of cases disproportionately impacted larger-sized counties, while the burden of deaths disproportionately impacted smaller counties. This may be partially due to multiple interacting social mechanisms, including a higher proportion of older adults who live in smaller counties. Moreover, these likely social mechanisms interacting with vaccinations and virus waves created a dynamic pattern whereby the rate and magnitude of infections and deaths were population- and time-dependent. Our results offer a novel perspective on the scaling dynamics of infectious diseases, highlighting how both the rate and magnitude of COVID-19 cases and deaths scale differently across counties. Population size and age structure are key factors in predicting disease burden. Our findings have practical implications, suggesting that scaling-informed public health policies could more effectively allocate resources and interventions to mitigate the impact of future epidemics across heterogeneous populations.

## 1. Introduction

The rapid global spread and persistence of the SARS-CoV-2 virus and disease (COVID-19) pandemic highlight the speed of interaction and magnitude of increasingly connected human populations in the twenty-first century. Despite substantial efforts to understand COVID-19 dynamics, there remains a gap in how disease burden scales across diverse populations of different sizes. The challenge of managing COVID-19 is compounded by spatial and temporal heterogeneity in disease dynamics [1,2], which vary significantly across regions with different population densities and demographic structures [3,4].

One promising approach to address this variability is allometric scaling, a quantitative framework traditionally used to study how biological characteristics change predictably with changes in the size of an organism or population (see [5] for an overview). Applied initially in ecology and physiology, allometric scaling has been extended to human populations to examine how various infrastructural, socio-economic, and biological attributes—such as disease prevalence—change predictably with population size in modern urban societies [6–11]. Early analysis of infectious disease scaling—such as analyses of AIDS cases in the United States and Brazil—revealed superlinear scaling relationships, where larger cities demonstrated disproportionately higher disease incidence [6,12]. These findings support the hypothesis that larger and more dense populations are disproportionately more affected by disease than those with smaller and less dense populations, likely due to a higher total number of contacts and speed of social interactions [13]. However, these studies focused primarily on stable, long-term infections and did not account for the acute temporal fluctuations characteristic of fast-spreading viral epidemics like COVID-19.

Recent scaling research on COVID-19 confirmed that early pandemic case growth rates scale superlinearly with city population size [14]. Yet, this work was largely limited to initial phases of the pandemic and urban centres, with limited exploration of dynamic scaling patterns across diverse demographic and geographic regions. Subsequent work in Brazil [15] and England and Wales [16] showed a temporal change in scaling exponents across the pandemic but not in relation to vaccines and variants. Given the United States’ disproportionately large share of global COVID-19 cases and deaths [17,18], a comprehensive scaling analysis that accounts for time-dependent shifts and demographic heterogeneity across variant waves is needed.

In this study, we address these gaps by examining allometric scaling relationships of COVID-19 cases, deaths, demographic cohorts and medical infrastructure across the pandemic, starting with the wild-type (original) virus and its first three major variants (alpha, delta, omicron) in the United States. By applying a dynamic scaling approach across counties, we uniquely assess how population size influences the rate and magnitude of COVID-19 outcomes over the first two years (2020−2022) of the pandemic. Unlike previous work that primarily focused on the growth of early cases, our study considers both cases and deaths, integrating demographic factors (e.g. age structure) and healthcare capacity (e.g. intensive care unit (ICU) beds) to provide a more rigorous assessment of scaling relations across urban–rural gradients. This approach allows us to capture how disease burden shifts from larger to smaller populations over time and assess potential underlying mechanisms, such as healthcare capacity and age distribution, which vary by population size.

## 2. Material and methods

### 2.1. Data

We used daily time-series data for COVID-19 cases and deaths, starting from 22 January 2020 (as day 1) and ending 31 March 2022 (as day 800), from the Johns Hopkins University Center for Systems Science and Engineering COVID-19 Data Repository [19]. These data included projected county-level population sizes in 2019 from 2010 United States Census Bureau data for all counties (3142), or county equivalents, in the United States. Data for county-level ICU hospital beds and the number of people aged 60 and older with respect to county total population were obtained from Kaiser Health News [20]. Only counties with one or more ICU hospital beds were included in the analysis (*n* = 1477). Finally, we used data on seven-day average cases and deaths from Our World in Data [21].

### 2.2. Model

We used a power function to evaluate the scaling relationship between COVID-19 cases, deaths, demographic cohorts, hospital ICU beds and county-level population size in the United States:


(2.1) $$Y\left(t\right)=Y_{0}N{\left(t\right)}^{\beta },$$

where *Y* is the given attribute for a county at time *t*, *N* is human population size as a measure of city size, *Y*_0_ is the normalization constant (intercept or elevation) and *β* is the exponent.

We linearized this equation to facilitate parameter estimation:


(2.2) $$\ln\left(Y\left(t\right)\right)=\ln\left(Y_{0}\right)+\beta \left(\ln N\left(t\right)\right).$$

**Figure 1.**
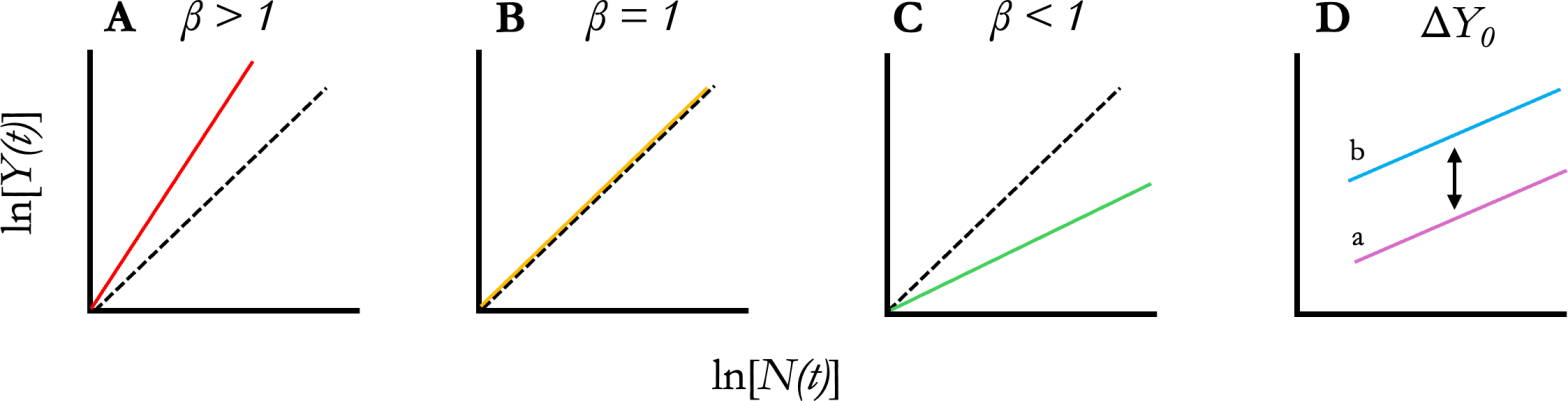
Hypothetical scaling relationships between population size as a function of time, *N*(*t*), and a population attribute as a function of time, *Y*(*t*). (A) Superlinear scaling, where *β* > 1. (B) Isometric, or linear scaling, where *β* = 1. (C) Sublinear scaling, where *β* < 1. (D) Two lines, *a* and *b,* with the same *β* but with different (Δ) elevations, *Y*_0_. Dashed line represents the 1:1 correspondence in (A–C).

In this form, the first term ln(*Y*_0_) reflects the expected magnitude of the attribute when the population size is 1 (i.e. ln(*Y*(*t*)) = ln(*Y*_0_) when ln(*N*(*t*)) = 0, implying that *N*(*t*) = 1 since ln(1) = 0). The *β* in the second term reflects the rate at which the attribute changes with county population size and is categorized among three classes of scaling behaviour: sublinear (*β* < 1), linear or isometric (*β* = 1) and superlinear (*β* > 1) (see also figure 1 for a conceptual overview of *β* and *Y*_0_). Parameters were estimated using the ordinary least squares (OLS) regression method, and 95% confidence intervals (CIs) for each parameter were computed from bootstrapped estimates of regression coefficients to partially correct for violations of the assumptions of normality and homoscedasticity (see electronic supplementary material for a complete description of method and testing assumptions).

### 2.3. Analysis

All analyses were conducted in R (v. 4.2.1) [22], and visualizations were made using the *ggplot2* (v. 3.5.1) [23], *ggpubr* (v. 0.6.0) [24] and *cowplot* (v. 1.1.2) [25] packages. We grouped cumulative COVID-19 cases and deaths in the United States according to the first four major virus waves: wild-type, alpha, delta and omicron. The wild-type wave corresponds to days 1−415 in the analysis. Regression analyses for wild-type cases and deaths start on day 50 and are reported daily until day 415 (11 March 2021). The COVID-19 alpha variant (B.1.1.7) became dominant in the United States on approximately 12 March 2021 (day 416 in our analysis) [26,27]. From that day onwards, we assumed that all new cumulative COVID-19 cases and deaths were attributed to ‘alpha’ in our analysis and calculated and reported new regression parameter estimates relative to the variant. The alpha wave corresponds to days 416−496 in the analysis. Regression analyses for alpha variant cases and deaths start on day 416 and are reported daily until day 496 (31 May 2021). The U.S. Centers for Disease Control and Prevention (CDC) reported that the COVID-19 delta variant (B.1.617.2) became dominant in the United States on 1 June 2021 (day 497 in our analysis). From that day onwards, we assumed that all new cumulative COVID-19 cases and deaths were attributed to ‘delta’ in our analysis. We calculated and reported new regression parameter estimates relative to the variant. The delta wave corresponds to days 497−689 in the analysis. Regression analyses for delta variant cases and deaths start on day 497 and are reported daily until day 689 (10 December 2021). The CDC reported that during the week of 11−18 December 2021 (days 690−697 in our analysis) the COVID-19 omicron variant (B.1.1.529) became dominant in the United States. From day 690 onwards, we assumed that all new cumulative COVID-19 cases and deaths were attributed to ‘omicron’ in our analysis. We calculated and reported new regression parameter estimates relative to the variant. The omicron wave corresponds to days 690−800 in the analysis. Regression analyses for omicron variant cases and deaths start on day 690 and are reported daily until day 800 (31 March 2022). Only counties with one or more cases or deaths were included in the analysis for each variant.

## 3. Results

Our analysis reveals distinct scaling behaviours for COVID-19 cases and deaths with county population size, characterized explicitly by superlinear scaling for cases and sublinear scaling for deaths. Superlinear scaling (*β* > 1) for cases indicates that larger counties experience disproportionately high numbers of cases, likely due to more frequent social interactions in dense populations. In contrast, sublinear scaling (*β* < 1) for deaths suggests that smaller counties bear a higher relative mortality burden, possibly due to limited healthcare resources and older population demographics. Our analysis also reveals that the magnitudes, ln(*Y*_0_), of COVID-19 cases and deaths are temporally dynamic, specifically changing within and among each wave.

**Figure 2.**
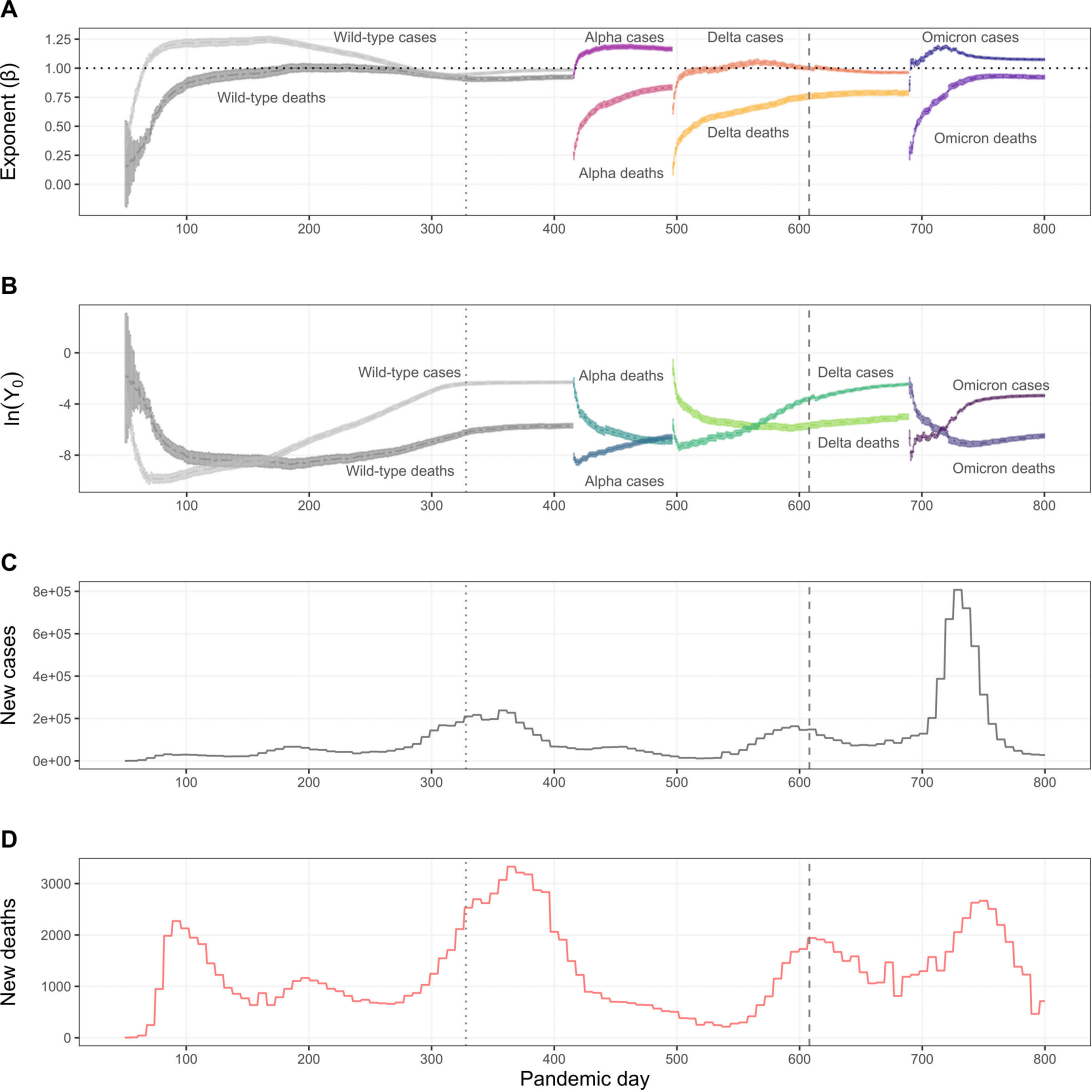
COVID-19 dynamics across the first two years of the pandemic (11 March 2020 (day 50)–31 March 2022 (day 800)). (A) Time-series change of scaling exponents (*β*) for cases and deaths, where shaded areas represent bootstrapped 95% CI. Dotted horizontal line indicates isometric scaling. (B) Time-series change of normalization constant (*Y*_0_) for cases and deaths. Shaded areas represent bootstrapped 95% CI. (C) Seven-day average of new cases. (D) Seven-day average of new deaths. Dotted vertical line in each panel indicates the start of COVID-19 vaccinations in the United States (day 328, 14 December 2020), and dashed vertical line indicates the start of the availability of the first COVID-19 booster vaccinations (day 608, 20 September 2021).

Figure 2 illustrates the time-dependent changes in scaling exponents (*β*, figure 2A) and normalization constants (ln(*Y*_0_), figure 2B) for COVID-19 cases and deaths as a function of county population size within and across each variant. Overall, *β* values for cases initially increase to a maximum for each variant (wild-type: 1.25, 95% CI [1.22, 1.27], day 167; alpha: 1.19, CI [1.17, 1.21], day 462; delta: 1.06, 95% CI [1.04, 1.08], day 566; omicron: 1.18, 95% CI [1.17, 1.20], day 720), and then slowly decrease thereafter. *β* values for deaths follow a similar pattern for each variant (wild-type: 1.0, 95% CI [0.97, 1.04], day 197; alpha: 0.83, CI [0.81, 0.86], day 496; delta: 0.79, 95% CI [0.77, 0.81], day 679; omicron: 0.93, 95% CI [0.92, 0.95], day 763). In terms of ln(*Y*_0_), the magnitude of cases and deaths initially declines then steadily rises over time within each variant wave. We also find that the expected magnitude of deaths is initially higher than the expected magnitude of cases for each wave and then reverses as the variants persist in the population. Seven-day average changes in cases (figure 2C) and deaths (figure 2D) accompany changes in *β* and ln(*Y*_0_). *R*^2^ values (electronic supplementary material, figure 1A) and the number of counties reporting at least one case or one death for each variant (electronic supplementary material, figure 1B) increase over the analysis.

**Figure 3.**
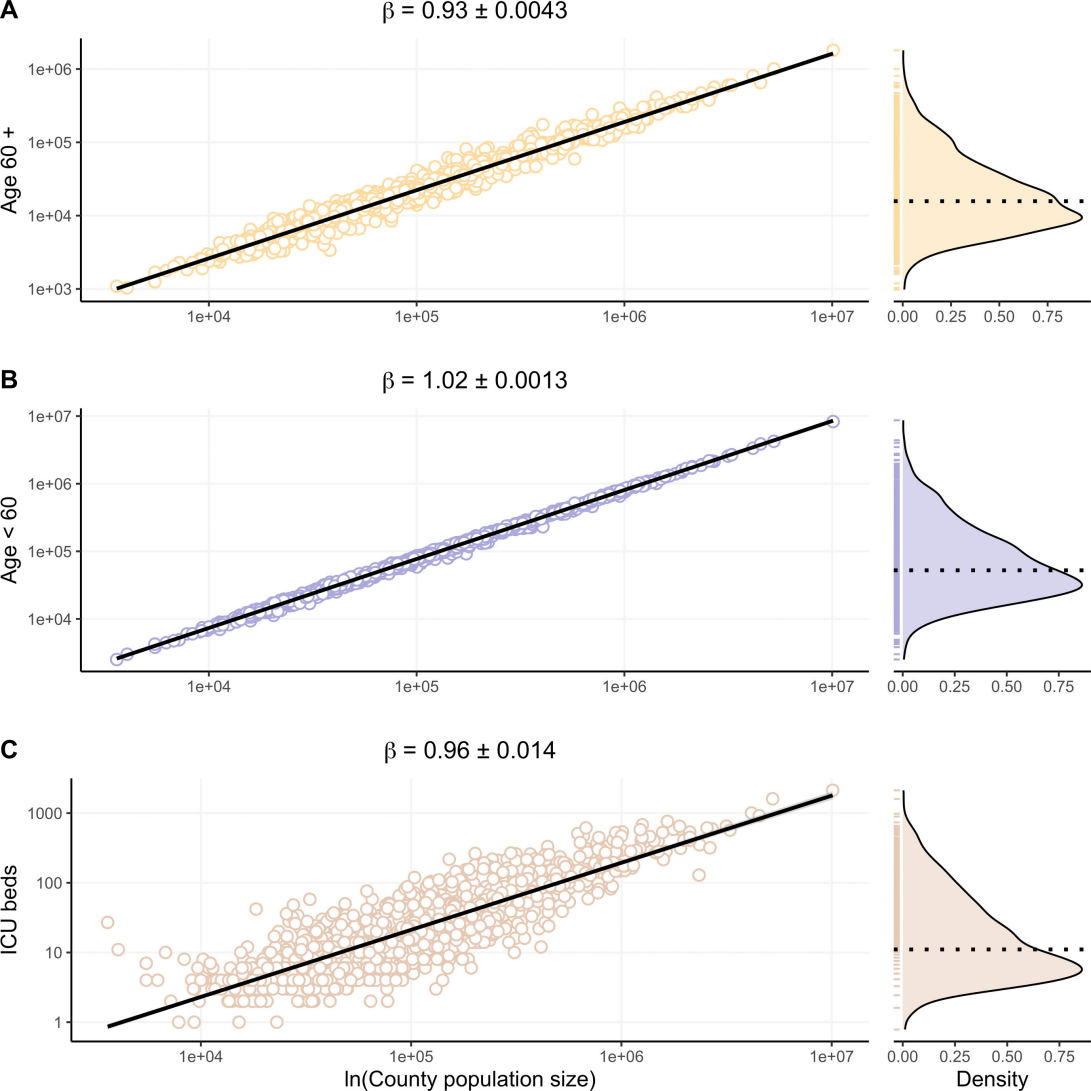
Allometric scaling of demographic cohorts and medical infrastructure. (A) Smaller population counties have proportionally more older adults (*β* < 1) than larger counties. (B) Younger populations are constant across counties (*β *~ 1). (C) Smaller-county populations have disproportionately more ICU beds (*β* < 1). Axes are natural log-transformed for each panel (A–C), and density distributions and their median values (dotted line) are shown to the right of each panel.

Additionally, our analyses also demonstrate a sublinear scaling between county population size and the county’s population aged 60 and older (*β* = 0.93 ± 0.0043 (s.e.); figure 3A), and linear scaling between county population size and the county’s population that is younger than 60 (*β* = 1.02 ± 0.0013 (s.e.); figure 3B). The scaling relationship between hospital ICU beds in a county and its population size is sublinear (*β* = 0.96 ± 0.014 (s.e.); figure 3C). Each of these results demonstrate left-skewed response variables, suggesting a wide range of larger values.

## 4. Discussion

Our findings reveal distinct scaling relationships for COVID-19 cases and deaths across US counties over the first two years of the pandemic. Specifically, we generally observe superlinear scaling for cases and sublinear scaling for deaths with county population size (figure 2; electronic supplementary material, figure S2). This distinction underscores a fundamental epidemiological insight: larger counties experience disproportionately high infection rates, whereas smaller counties bear a heavier mortality burden relative to their size. This is demonstrated in the observation that the scaling exponent for cases is predominantly superlinear (>1) in each pandemic wave, with a trend towards isometry (=1). In contrast, the scaling exponent for deaths is consistently sublinear (<1) in each pandemic wave. Intriguingly, we also observe that the expected magnitude (normalization constant) of deaths is initially higher than that of cases in each wave, which might be partially explained by the virus’ increased virulence as it mutated over time [28,29], and that this pattern is reversed as the virus and its variants establish, possibly due to subsequent population-level immunity after initial exposure. Together, we observe a dynamic change in both the scaling exponent and normalization constant over time within and across each wave. This temporal variability indicates that the rate and magnitude of infections and deaths is population- and time-dependent, often starting in larger, ‘core’ populations and spreading to smaller ones [1,30,31].

These patterns likely reflect the interplay of social, demographic and healthcare factors, highlighting the need for public health interventions tailored to population characteristics. For example, smaller, more rural population counties have been shown to have a larger healthcare system burden due to fewer available medical resources, such as infectious disease specialists [32], as well as a higher proportion of older adults (figure 3A) [30] that also have higher mortality rates from COVID-19 [33,34]. Furthermore, although the availability of vaccines likely averted many deaths in the United States [35] (e.g. figure 2D; note the peak and subsequent decline in seven-day average deaths during the delta variant wave after the availability of the first booster vaccination, day 608), vaccine uptake was significantly lower among rural versus urban counties [36]. This might partially explain the sublinear scaling behaviour for deaths across variants. Moreover, protective health measures in counties of different sizes may also be related to experience of disease burden [37] and/or political orientation [36,38]. To address these public health disparities, policy and management strategies in the United States should consider how to scale both medical resources (e.g. hospital beds, medical professionals, antiviral medication, vaccinations) and social resources (e.g. targeted information campaigns) to prevent or reduce mortality of vulnerable groups [30,39]. This might be achieved by developing resource allocation models explicitly informed by infrastructural and demographic scaling relations.

Globally, our results show mixed agreement with patterns observed in other countries. For example, Brazil demonstrated an opposite trend with respect to our scaling results for cases (sublinear to linear), deaths (superlinear) and ICU beds (superlinear) over the first four months of the initial wave, but similar demographic scaling relative to the United States [15]. In contrast, scaling patterns for cases and deaths in England and Wales were largely consistent with ours, reporting superlinear scaling for cases and sublinear scaling for deaths over the first year of the pandemic [16]. Interestingly, England and Wales also demonstrated similar demographic scaling to the United States [40]. Together, these patterns indicate the need to understand regional variation in the multiple interacting mechanisms that factor into the burden of COVID-19 infection and mortality across the globe.

Our results are not without limitations. We used fixed county-level population sizes, which may not fully account for short-term and long-term spatial redistribution of populations during the pandemic. Migration from urban to rural areas and movement among counties affected population dynamics in the United States [41,42]. These shifts could have introduced uncertainty in estimating infection and death rates over time. We also acknowledge the potential influence of time lags. Delays between initial infections, reporting, and death may have affected our parameter estimates (e.g. [15]). These lags could have led to underestimation or overestimation of scaling coefficients for certain variants. To improve the robustness of our analyses, we implemented bootstrap methods to estimate model coefficients (see electronic supplementary material). However, our results should be considered alongside the limitations of our model choice. Future work should address common challenges (e.g. zero-inflated data, nonlinearity, heteroscedasticity) in allometric scaling studies [43–45]. Despite these potential limitations, our study provides valuable insights into scaling dynamics, highlighting the complex interplay of population size, infection rates and mortality during the COVID-19 pandemic. Further refinement of scaling models will help to improve predictive accuracy and support better-informed public health interventions.

Nevertheless, the allometric scaling approach that we have taken here towards human epidemiology offers several critical insights and advantages. First, our results confirm and add nuance to the well-established scaling predictions for how disease dynamics operate within and among human populations, particularly at the county level [46]. In this case, we demonstrated that infectious disease scales superlinearly within populations but that components of the disease, such as infection versus mortality, may behave differently due to multiple mechanisms that likely interact. Second, previous scaling approaches have generally considered only the rate of change (*β*) of an attribute, but here, we have additionally investigated the magnitude by which it changes (ln(*Y*_0_)), adding further insight into the importance of the parameter to disease dynamics and its application in biology more broadly [47]. Finally, while consistent with other findings [30,34], our approach has the advantage of requiring minimal data and analytic complexity. Our method enables rapid assessments of evolving disease dynamics, making it useful for policy makers and medical staff who may quickly become overburdened during infection spikes. Allometric scaling has proven to be a valuable framework for understanding COVID-19 dynamics across diverse populations. Continued efforts to advance scaling as both a scientific tool and diagnostic method in epidemiology will enhance its relevance and effectiveness in future research and interventions [48,49]. Such scaling insights can guide public health strategies to better prepare for and respond to future pandemics.

## Data Availability

Data and associated code are available in Zenodo [50]. Supplementary material is available online [51].

## References

[1] Kortessis N, Simon MW, Barfield M, Glass GE, Singer BH, Holt RD. 2020 The interplay of movement and spatiotemporal variation in transmission degrades pandemic control. Proc. Natl Acad. Sci. USA **117**, 30104–30106. (10.1073/pnas.2018286117) 33172993 PMC7720174

[2] Tuladhar R, Grigolini P, Santamaria F. 2022 The allometric propagation of COVID-19 is explained by human travel. Infect. Dis. Model. **7**, 122–133. (10.1016/j.idm.2021.12.003) 34926874 PMC8670009

[3] Sy KTL, White LF, Nichols BE. 2021 Population density and basic reproductive number of COVID-19 across United States counties. PLoS ONE **16**, e0249271. (10.1371/journal.pone.0249271) 33882054 PMC8059825

[4] Monod M *et al*. 2021 Age groups that sustain resurging COVID-19 epidemics in the United States. Science **371**, eabe8372. (10.1126/science.abe8372) 33531384 PMC8101272

[5] Savage VM, Deeds EJ, Fontana W. 2008 Sizing up allometric scaling theory. PLoS Comput. Biol. **4**, e1000171. (10.1371/journal.pcbi.1000171) 18787686 PMC2518954

[6] Bettencourt LMA, Lobo J, Helbing D, Kühnert C, West GB. 2007 Growth, innovation, scaling, and the pace of life in cities. Proc. Natl Acad. Sci. USA **104**, 7301–7306. (10.1073/pnas.0610172104) 17438298 PMC1852329

[7] Bettencourt LMA. 2013 The origins of scaling in cities. Science **340**, 1438–1441. (10.1126/science.1235823) 23788793

[8] Burger JR, Weinberger VP, Marquet PA. 2017 Extra-metabolic energy use and the rise in human hyper-density. Sci. Rep. **7**, 43869. (10.1038/srep43869) 28252010 PMC5333137

[9] Bettencourt LM. 2021 Introduction to urban science: evidence and theory of cities as complex systems. Cambridge, MA: MIT Press.

[10] Uchida K, Blakey RV, Burger JR, Cooper DS, Niesner CA, Blumstein DT. 2021 Urban biodiversity and the importance of scale. Trends Ecol. Evol. **36**, 123–131. (10.1016/j.tree.2020.10.011) 33168154

[11] Burger JR *et al*. 2022 Global city densities: re-examining urban scaling theory. Front. Conserv. Sci. **3**, 879934. (10.3389/fcosc.2022.879934)

[12] Antonio FJ, de Picoli S, Teixeira JJV, Mendes R dos S. 2014 Growth patterns and scaling laws governing AIDS epidemic in Brazilian cities. PLoS ONE **9**, e111015. (10.1371/journal.pone.0111015) 25340796 PMC4207789

[13] Schläpfer M, Bettencourt LMA, Grauwin S, Raschke M, Claxton R, Smoreda Z, West GB, Ratti C. 2014 The scaling of human interactions with city size. J. R. Soc. Interface **11**, 20130789. (10.1098/rsif.2013.0789) 24990287 PMC4233681

[14] Stier AJ, Berman MG, Bettencourt LMA. 2021 Early pandemic COVID-19 case growth rates increase with city size. Npj Urban Sustain. **1**, 1. (10.1038/s42949-021-00030-0)

[15] Ribeiro HV, Sunahara AS, Sutton J, Perc M, Hanley QS. 2020 City size and the spreading of COVID-19 in Brazil. PLoS ONE **15**, e0239699. (10.1371/journal.pone.0239699) 32966344 PMC7510961

[16] Sutton J, Shahtahmassebi G, Ribeiro HV, Hanley QS. 2022 Population density and spreading of COVID-19 in England and Wales. PLoS ONE **17**, e0261725. (10.1371/journal.pone.0261725) 35358202 PMC8970409

[17] Msemburi W, Karlinsky A, Knutson V, Aleshin-Guendel S, Chatterji S, Wakefield J. 2023 The WHO estimates of excess mortality associated with the COVID-19 pandemic. Nature **613**, 130–137. (10.1038/s41586-022-05522-2) 36517599 PMC9812776

[18] Paglino E *et al*. 2023 Monthly excess mortality across counties in the United States during the COVID-19 pandemic, March 2020 to February 2022. Sci. Adv. **9**, eadf9742. (10.1126/sciadv.adf9742) 37352359 PMC10289647

[19] Dong E *et al*. 2022 The Johns Hopkins University Center for Systems Science and Engineering COVID-19 dashboard: data collection process, challenges faced, and lessons learned. Lancet Infect. Dis. **22**, e370–e376. (10.1016/S1473-3099(22)00434-0) 36057267 PMC9432867

[20] Schulte F, Lucas E, Rau J, Szabo L, Hancock J. 2020 Millions of older Americans live in counties with no ICU beds as pandemic intensifies. KFF Health News. See https://kffhealthnews.org/news/as-coronavirus-spreads-widely-millions-of-older-americans-live-in-counties-with-no-icu-beds/#lookup.

[21] Mathieu E. 2020 Coronavirus pandemic (COVID-19). See https://ourworldindata.org/coronavirus.

[22] R Core Team. 2022 R: a language and environment for statistical computing (v. 4.2.1). Vienna, Austria: R Foundation for Statistical Computing. See https://www.R-project.org/.

[23] Wickham H. 2011 ggplot2. WIREs Comput. Stats. **3**, 180–185. (10.1002/wics.147)

[24] Kassambara A. 2023 ggpubr: ‘ggplot2’ based publication ready plots. R package version 0.6.0. See https://CRAN.R-project.org/package=ggpubr.

[25] Wilke C. 2023 cowplot: streamlined plot theme and plot annotations for ‘ggplot2’. R package version 1.1.2. See https://CRAN.R-project.org/package=cowplot.

[26] Bolze A *et al*. 2022 SARS-CoV-2 variant delta rapidly displaced variant alpha in the United States and led to higher viral loads. Cell Rep. Med. **3**, 100564. (10.1016/j.xcrm.2022.100564) 35474739 PMC8922438

[27] Paul P *et al*. 2021 Genomic surveillance for SARS-CoV-2 variants circulating in the United States, December 2020-May 2021. MMWR Morb. Mortal. Wkly Rep. **70**, 846–850. (10.15585/mmwr.mm7023a3) 34111060 PMC8191868

[28] Andre M *et al*. 2023 From alpha to omicron: how different variants of concern of the SARS-coronavirus-2 impacted the world. Biology **12**, 1267. (10.3390/biology12091267) 37759666 PMC10525159

[29] Fisman DN, Tuite AR. 2021 Evaluation of the relative virulence of novel SARS-CoV-2 variants: a retrospective cohort study in Ontario, Canada. CMAJ **193**, E1619–E1625. (10.1503/cmaj.211248) 34610919 PMC8562985

[30] Miller IF, Becker AD, Grenfell BT, Metcalf CJE. 2020 Disease and healthcare burden of COVID-19 in the United States. Nat. Med. **26**, 1212–1217. (10.1038/s41591-020-0952-y) 32546823

[31] Grenfell BT, Bjørnstad ON, Kappey J. 2001 Travelling waves and spatial hierarchies in measles epidemics. Nature **414**, 716–723. (10.1038/414716a) 11742391

[32] Walensky RP, McQuillen DP, Shahbazi S, Goodson JD. 2020 Where is the ID in COVID-19? Ann. Intern. Med. **173**, 587–589. (10.7326/m20-2684) 32491920 PMC7277486

[33] Sasson I. 2021 Age and COVID-19 mortality. Demogr. Res. **44**, 379–396. (10.4054/DemRes.2021.44.16)

[34] Hamidi S, Sabouri S, Ewing R. 2020 Does density aggravate the COVID-19 pandemic? J. Am. Plan. Assoc. **86**, 495–509. (10.1080/01944363.2020.1777891)

[35] Watson OJ, Barnsley G, Toor J, Hogan AB, Winskill P, Ghani AC. 2022 Global impact of the first year of COVID-19 vaccination: a mathematical modelling study. Lancet Infect. Dis. **22**, 1293–1302. (10.1016/s1473-3099(22)00320-6) 35753318 PMC9225255

[36] Sun Y, Monnat SM. 2022 Rural‐urban and within‐rural differences in COVID‐19 vaccination rates. J. Rural Health **38**, 916–922. (10.1111/jrh.12625) 34555222 PMC8661570

[37] Angelopoulos K, Stewart G, Mancy R. 2023 Local infectious disease experience influences vaccine refusal rates: a natural experiment. Proc. R. Soc. B **290**, 20221986. (10.1098/rspb.2022.1986) PMC9890117 36722077

[38] Gonzalez KE, James R, Bjorklund ET, Hill TD. 2021 Conservatism and infrequent mask usage: a study of US counties during the novel coronavirus (COVID‐19) pandemic. Soc. Sci. Q. **102**, 2368–2382. (10.1111/ssqu.13025) 34908612 PMC8661919

[39] Mena GE, Martinez PP, Mahmud AS, Marquet PA, Buckee CO, Santillana M. 2021 Socioeconomic status determines COVID-19 incidence and related mortality in Santiago, Chile. Science **372**, g5298. (10.1126/science.abg5298) PMC8158961 33906968

[40] Sutton J, Shahtahmassebi G, Ribeiro HV, Hanley QS. 2020 Rural–urban scaling of age, mortality, crime and property reveals a loss of expected self-similar behaviour. Sci. Rep. **10**, 16863. (10.1038/s41598-020-74015-x) 33033349 PMC7545192

[41] Yilmazkuday H. 2020 COVID-19 spread and inter-county travel: daily evidence from the US. Transp. Res. Interdiscip. Perspect. **8**, 100244. (10.1016/j.trip.2020.100244) 34173479 PMC7580684

[42] Ramani A, Bloom N. 2021 The donut effect of COVID-19 on cities. NBER Working Paper, no. 28876. Cambridge, MA: National Bureau of Economic Research. (10.3386/w28876)

[43] Leitão JC, Miotto JM, Gerlach M, Altmann EG. 2016 Is this scaling nonlinear? R. Soc. Open Sci. **3**, 150649. (10.1098/rsos.150649) 27493764 PMC4968456

[44] Finance O, Cottineau C. 2019 Are the absent always wrong? Dealing with zero values in urban scaling. Environ. Plan. B **46**, 1663–1677. (10.1177/2399808318785634)

[45] Sutton J, Shahtahmassebi G, Hanley QS, Ribeiro HV. 2024 A heteroscedastic Bayesian generalized logistic regression model with application to scaling problems. Chaos Solitons Fractals **182**, 114787. (10.1016/j.chaos.2024.114787)

[46] Ives AR, Bozzuto C. 2021 Estimating and explaining the spread of COVID-19 at the county level in the USA. Commun. Biol. **4**, 60. (10.1038/s42003-020-01609-6) 33402722 PMC7785728

[47] Niklas KJ, Hammond ST. 2019 On the interpretation of the normalization constant in the scaling equation. Front. Ecol. Evol. **6**, 212. (10.3389/fevo.2018.00212)

[48] Brummer AB, Savage VM. 2021 Cancer as a model system for testing metabolic scaling theory. Front. Ecol. Evol. **9**, 691830. (10.3389/fevo.2021.691830)

[49] Loureiro NA, Neto CR, Sutton J, Perc M, Ribeiro HV. 2025 Impact of inter-city interactions on disease scaling. Sci. Rep. **15**, 498. (10.1038/s41598-024-84252-z) 39748086 PMC11696764

[50] Cruz AR, Enquist BJ, Burger JR. 2025 Data from: Scaling COVID-19 rates with population size in the United States. Zenodo. (10.5281/zenodo.14956993) PMC11937915 40134356

[51] Cruz AR, Enquist B, Burger R. 2025 Supplementary material from: Scaling COVID-19 rates with population size in the United States. Figshare. (10.6084/m9.figshare.c.7713150) PMC11937915 40134356

